# The efficacy of whole-course nursing on malignant bone and soft tumors of lower limbs: A randomized study

**DOI:** 10.1097/MD.0000000000049172

**Published:** 2026-08-07

**Authors:** Linlin Wang, Wen Tian, Jing Jing, Ming Yin, Yanna Sun

**Affiliations:** a Department of Bone and Soft Tissue Sarcoma, The Affiliated Cancer Hospital of Zhengzhou University and Henan Cancer Hospital, Zhengzhou, China; b School of Public Health, Zhengzhou University, Henan, China.

**Keywords:** bone and soft tumor, randomized controlled study, rehabilitation, usual nursing, whole-course nursing

## Abstract

Patients with malignant bone and soft tissue tumors of the lower limbs frequently encounter substantial postoperative pain, delayed functional recovery, and adverse psychological reactions. Conventional nursing care may not adequately address these complex needs during the perioperative period. This study aimed to assess the efficacy of a comprehensive whole-course nursing model - encompassing preoperative education, perioperative care, postoperative rehabilitation, and psychological support - in enhancing clinical outcomes for patients undergoing limb salvage surgery. In this randomized controlled trial, 116 eligible patients were recruited and randomly allocated into either a whole-course nursing group or a routine care group. Clinical endpoints included pain intensity (VAS), lower limb function (LEFS), emotional status (HAMA), patient satisfaction (HPOI and nursing satisfaction scores), and quality of life (Karnofsky index). Data were analyzed using independent t-tests and Cox proportional hazards regression analysis. Compared with routine care, the whole-course nursing group demonstrated significantly reduced pain scores at 24 hours and 48 hours post-surgery (*P* = .046 and *P* < .001, respectively), improved lower limb functional outcomes (*P* < .001), diminished adverse emotions (*P* = .002), and enhanced quality of life scores (*P* = .003). Patient satisfaction regarding both pain management and overall nursing care was also markedly higher (*P* < .001 and *P* = .001). Cox regression analysis identified the nursing model (*P* = .017), postoperative recovery (*P* = .021), and quality of life (*P* = .028) as independent prognostic factors. The implementation of a whole-course nursing model significantly enhances pain control, emotional well-being, functional recovery, and overall quality of life in patients with malignant bone and soft tissue tumors.

## 1. Introduction

The incidence of primary malignant bone tumors is low, accounting for approximately 0.2% of all malignant tumors^[1,2]^; however, the prognosis of patients with malignant bone tumors is poor because of their high malignancy and aggressiveness.^[3]^ Advancements have led to increasingly tailored and effective treatments for malignant bone tumors. The principle of clinical treatment is limb preservation, which ensures the sound of limbs and motor and sensory function and reduces the feasibility of complications to a certain extent.^[4]^

Neoadjuvant chemotherapy has become the primary treatment mode for malignant bone tumors.^[5]^ However, even after successful treatment, patients face the threat of chemotherapy, surgical stress, tumor recurrence, and even metastasis, as well as a longer hospital stay and high medical costs, which result in greater economic and psychological pressure on patients, making them susceptible to anxiety if they are diagnosed. Moreover, as patients need chemotherapy shortly after limb salvage surgery, professional rehabilitation training cannot be performed in a timely manner to explore the best opportunity for limb function recovery. To date, exploring effective nursing measures to improve lower limb function and change anxiety and other adverse emotions in patients with bone tumors is difficult.

Whole-course nursing, a new nursing model, includes both preoperative guidance for limb rehabilitation training and postoperative continuous rehabilitation training, as well as whole-course psychological guidance with the participation of psychological counselors.^[6]^ Simultaneously with rehabilitation guidance for patients, rehabilitation nursing interferes with patients’ negative emotions, which plays a better role in eliminating anxiety and promoting treatment cooperation.^[7]^

This randomized controlled study evaluated the effectiveness of a whole-course nursing program for patients with malignant bone and soft tumors of the lower limbs postoperatively. We hypothesized that compared with the control group receiving normal nursing, the experimental group receiving whole-course nursing would provide positive feedback on the outcomes of physical function, pain, quality of life, satisfaction with nursing, and levels of negative emotions.

## 2. Materials and methods

### 2.1. Study design

This randomized study was conducted from January 2021 to June 2023 and was approved by the Ethical Committee of Henan Cancer Hospital (No. 2022-200-001). All participants provided written informed consent before their participation in the study.

### 2.2. Sample size

**Figure 1.**
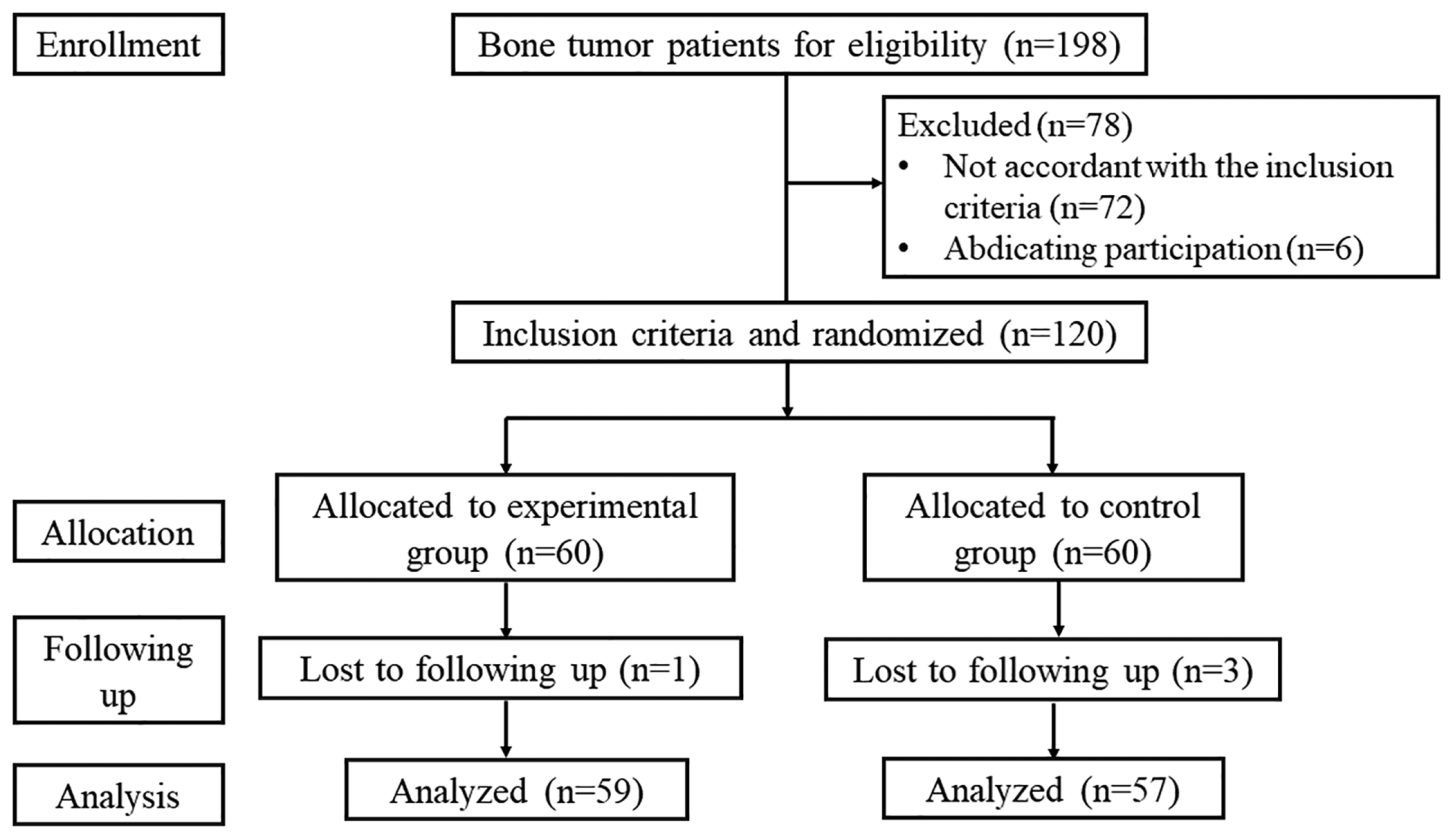
Flowchart showing the process of participant recruitment, randomization, allocation, follow-up, and final analysis.

The sample size was estimated using PASS software (version 11.0), assuming a between-group difference of 1.5 in VAS scores with a standard deviation of 2.5. A sample size of 122 patients was required for this study, with a statistical power of 80% and a 2-sided significance level of 0.05. Considering an attrition rate of 10%, 110 patients were required. Based on the inclusion criteria, 120 patients were enrolled in our study, and 60 patients were randomly assigned to the control and experimental groups. During follow-up, 4 patients were lost to follow-up, and 116 patients were analyzed systematically (Fig. 1).

### 2.3. Participant recruitment

The patients were recruited from Henan Cancer Hospital in Henan Province, a 3A hospital in China, with high-quality diagnoses, treatments, and services.

The inclusion criteria of this study were as follows: no amputation before treatment and diagnosis of primary malignant bone tumor of the lower limb by MRI (magnetic resonance imaging) or CT (computed tomography). The exclusion criteria were as follows: amputation before research, and other malignant tumors, immune deficiency, or hematological diseases.

The patients were assessed by experienced nurses and enrolled in the study if they met the criteria. Patients were informed of the research protocol in detail and signed an informed consent form.

### 2.4. Randomization

Block randomization with a fixed block size of 4 was employed to ensure balanced group allocation throughout the recruitment period. The randomization sequence was generated by an independent epidemiologist using a computer-generated random number list. Group assignments were enclosed in sealed, opaque, and sequentially numbered envelopes to maintain allocation concealment. The outcome assessors, who were responsible for data collection and evaluation of primary endpoints, including pain score, limb function, emotional status, and quality of life, were blinded to the group allocations. Due to the inherent nature of the nursing intervention, blinding of participants and care providers was not feasible. However, outcome assessments were carried out independently by trained evaluators who were unaware of the treatment assignments in order to minimize potential measurement bias.

### 2.5. Interventions

The program was developed by experienced clinicians and nurses. The control group adopted usual nursing, which included psychiatric and chemotherapy nursing, preoperative nursing, postoperative rehabilitation nursing, and discharge nursing instruction (Table 1). Psychiatric and chemotherapy nursing refers to eliminating concerns and stabilizing the emotions of patients according to their different psychological characteristics. Meanwhile, nurses were comprehensively used to educate patients about chemotherapy, observe side effects, and actively prevent and deal with them to assist patients in completing chemotherapy successfully. Preoperatively, patients were guided by deep breathing, cough training, activity of the affected limb, and use of toilets in bed. After the operation, vital signs, limb blood circulation, sensorimotor function, and fluid drainage were closely observed. The wound dressing was kept clean and dry, and the drainage tube was kept fixed and unobstructed. Nutrition and hydration of patients were ensured to reduce the incidence of complications. At 1 to 5 days, passive activities were performed to assist joint flexion and extension activities. Fifteen to 60 days after surgery, gradual walking with weight was used to further strengthen muscle strength and gradually regain the ability to live daily life. Hospital discharge guidance was provided to patients before discharge, including continued functional exercise, regular review and follow-up, and return to the hospital to continue chemotherapy on time.

**Table 1:** The details of usual nursing and the whole-course rehabilitation nursing.

		Usual nursing	Whole-course rehabilitation nursing
Contents	Psychiatric and chemotherapy nursing	(1) To stabilize emotions and create a congenial environment for recuperation, strive for the cooperation of patients and family members, eliminate patients concerns, enhance the confidence of treatment. (2) To evaluate the conditions and comprehensively educate chemotherapy knowledge, observe chemotherapy side effects, actively prevent and deal with them. (3) To clarify the liver and kidney functions and bone marrow hematopoietic functions of patients, observe whether patients have potential infection lesions, etc	(1) To check the mastery of the patient twice per day, continuing to guide the patients until complete mastery. (2) To help patients to fully understand and accept the malignant tumor and successfully through the psychological crisis, psychological counselors participate in the whole process to fully assess the psychological state, physical function and rehabilitation needs after admission.
	Preoperative rehabilitation nursing	(1) To perform deep breathing and cough training, and use toilets in bed. (2) To guide the activity of the affected limb to prevent pathological fracture. (3) To assist in improving preoperative examination and preparation.	(1) To fully inform the purpose and significance of postoperative rehabilitation exercise since the patients were admitted to hospital. To auxiliary understand the importance of rehabilitation exercise of patients and relatives through detailed case explanations. To establish the adequate psychological preparation to actively respond with the long-term postoperative functional rehabilitation exercise. (2) According to the lesion site of patients, to guide and teach patients functional exercise methods in different postoperative periods.
	Postoperative nursing	(1) To observe closely the vital signs, limb blood circulation, sensorimotor function and drainage fluid. (2) To keep wound dressing clean and dry, drainage tube fixed and unobstructed. (3) To strengthen basic nursing and reduce the occurrence of complications.	(1) Since the patients postoperative awakening under general anesthesia or continuous epidural anesthesia, the recovery of limb sensation and motor function, to closely observe the condition, and give the patients passive limb activity to promote lower limb blood circulation and avoid adhesion, muscle atrophy and other complications as soon as possible. (2) In the process of rehabilitation training, to pay attention to whether intensity and amount of exercises are appropriate, and discuss with the attending doctors to understand the special requirements of postoperative rehabilitation exercise of patients, appropriate adjustment of rehabilitation exercise plan, and strictly ensure the completion of training.
	Postoperative rehabilitation nursing	(1) 1–5 d after surgery: to carry out passive activity to assist joint flexion and extension activities. And active training to pull contracture tissue, avoid adhesion, promote lower limb blood circulation, prevent deep vein thrombosis and embolism. (2) 5–14 d after surgery: to carry out active activities, such as lifting knees on the thigh, lateral knee flexion and extension without gravity on the affected limb, to restore joint function and strengthen muscle strength. (3) 15–60 d after surgery: to gradually walking with weight, and carry out unassisted functional exercises. After the suture removal, to massage the scar site to soften the scar, further strengthen the muscle strength and increase the balance, and gradually restore the ability of daily life.	
	Discharge nursing instruction	To inform discharge instruction, including methods of continuing functional exercise, regular review and follow-up, returning to hospital on time to continue chemotherapy.	(1) To do a good job of patient follow-up work after discharge. (2) To set up a continuous rehabilitation team in the ward. The members of the team should be the third-level physicians and experienced nurses. (3) Following up could be conducted by telephone or Internet. To control the progress of rehabilitation exercise of the patients, and carry out limb function assessment, and provide relevant guidance and suggestions.

The experimental group received whole-course nursing, which was refined and comprehensive in addition to routine nursing, to take appropriate measures to promote rehabilitation based on the theory of nursing management model to solve the rising medical costs in the 1980s in America (Léon Navarro, García, & García, 2014). Psychological state, physical function, and rehabilitation needs of psychological counselors throughout the course were assessed (Table 1). Psychological changes and potential negative emotions were promptly identified and addressed in a timely manner. Upon admission, patients were fully informed about the purpose and significance of postoperative rehabilitation exercises, thereby enhancing their awareness of the importance of such activities. Adequate psychological preparation was established to enable patients to actively cope with long-term and demanding postoperative functional rehabilitation. Based on the location of the lesion, patients were guided and instructed on appropriate functional exercises during different postoperative stages. Responsible nurses followed up with patients twice daily to assess their proficiency in performing these exercises. Postoperatively, patients’ conditions were closely and comprehensively monitored, and passive limb movements were initiated after stabilization to promote lower limb circulation and prevent complications such as adhesion and muscle atrophy. Throughout the rehabilitation process, the intensity and volume of exercise were carefully considered to ensure strict adherence to prescribed rehabilitation protocols. Follow-up was conducted after discharge to monitor ongoing recovery.

To ensure the consistency and high quality of full-course nursing interventions across all participants, a set of standardized protocols and supervisory mechanisms was established. Prior to the commencement of the study, all participating nurses underwent centralized training based on a unified intervention framework. This framework clearly outlined the timeline, operational procedures, and follow-up management strategies for each stage of care. Key components included preoperative education, phased postoperative rehabilitation guidance, and structured psychological support. During the implementation phase, nurses maintained daily records of care delivery. Following discharge, a multidisciplinary rehabilitation team, comprising experienced nurses and attending physicians, conducted regular patient follow-ups via telephone or online communication platforms. Structured assessment forms were used during these follow-ups to systematically evaluate limb function recovery, adherence to the rehabilitation plan, emotional well-being, and the emergence of any new complications. Real-time feedback was provided, along with individualized guidance, to ensure that each patient’s functional training progressed according to plan. These measures ensured that all patients received comprehensive, standardized, and continuously monitored care throughout the perioperative and rehabilitation periods. Periodic reviews were conducted to ensure strict adherence to the intervention framework, with any deviations documented and analyzed for quality improvement purposes.

### 2.6. Measure indicators

After collection, the data were checked and recorded by 2 epidemiologists who were not involved in this study. All validated outcome measures were assessed at predefined standardized time points. Specifically, the Visual Analogue Scale (VAS) was evaluated preoperatively, as well as at 24 hours and 48 hours postoperatively. The Hip Outcome Score (HOS), patient satisfaction with care, and the Hamilton Anxiety Scale (HAMA) were assessed at the time of hospital discharge. The Lower Extremity Functional Scale (LEFS) score was recorded both preoperatively and at discharge. In addition, adverse events and quality of life were reassessed during the 6-month postoperative follow-up visit (Table S1, Supplemental Digital Content 1).

#### 2.6.1. Pain score

The pain scores were measured using the VAS score. This scale consists of 4 items, ranging from 0 “not at all, ≤3 “slight pain, 4 to 6 “pain and tolerable to 10 “pain but unbearable.”^[8]^

#### 2.6.2. Lower extremity functional score

The lower extremity functional scale (LEFS) was used to measure functional scores of the lower extremities, including functional activities, stepping ability, limb pain, and gait changes. The full score of each item was 25, with a lower score for limb pain indicating greater improvement and a higher score for other items indicating further improvement.

#### 2.6.3. Satisfaction with pain relief

The HPOI (Houston Pain Outcome Instrument) was used to assess the level of satisfaction with the postoperative pain relief. The scores range from 0 to 10, with higher scores indicating higher satisfaction.

#### 2.6.4. Satisfaction with nursing

Patient satisfaction was evaluated after nursing. The nursing satisfaction questionnaire designed by our hospital was used to collect the scores (Supplementary Questionnaire, Supplemental Digital Content 2). A total of 10 items were included, including ward environment, health education, nursing staff service skills, service attitude, nursing responsibility, nursing timeliness, nursing initiative, nursing humanization, nurse-patient communication, dressing, and appearance, with a total score of 100, including satisfaction (80–100 points), general (50–79 points), dissatisfaction (0–49 points).

#### 2.6.5. Adverse emotion assessment

The Hamilton Anxiety Scale (HAMA) was used to assess the level of adverse emotions. Joint inspection and independent scoring were also performed. All items were scored on a 5-point scale ranging from 0 “asymptomatic,” 1 “light,” 2 “medium,” 3 “heavy,” and 4 “extremely heavy.”

#### 2.6.6. Quality of life score

The Karnofsky evaluation index was used to score the life quality of patients ranging from 100 “normal,” 90 “ability to perform normal activities with mild signs and symptoms,” 80 “struggling to perform normal activities,” 70 “unable to maintain a normal life and work,” 60 “mostly self-care,” 50 “often in need of care,” 40 “need special care and help,” 30 “seriously unable to take care of themselves,” 20 “seriously ill with requiring hospitalization and active supportive care,” 10 “critical danger,” to 0 “death.” A higher score indicates better health, and a lower score indicates worse health.

#### 2.6.7. Postoperative complications

Adverse postoperative complications such as wound infection, bleeding, and deep vein thrombosis have received close attention.

### 2.7. Statistical analysis

The SPSS software (version 21.0) was used to process the data. The percentage and frequency were described using the chi-square test. In accordance with normal distribution, measurement data were described as mean ± standard deviation and analyzed using an independent sample *t* test. If the data did not conform to a normal distribution, they were analyzed using the rank test. To address the potential inflation of type I error rates arising from multiple comparisons, the false discovery rate (FDR) correction procedure introduced by Benjamini and Hochberg was employed where appropriate, using an adjusted significance level of *q* < 0.05. *P* < .05 was defined as a statistically significant difference.

## 3. Results

### 3.1. Participants characteristics

A total of 116 patients were enrolled in this study for eligibility, of which 198 were excluded and 4 were lost to follow-up (Fig. 1). Ultimately, 59 patients were allocated to the control group and 57 patients were allocated to the experimental group, who had completed all the processes and collected all data.

The patient characteristics are listed in Table 2. The mean ages of the experimental and control group were 25.474 ± 20.776 and 25.068 ± 19.560 years, respectively. The BMI in the experimental group was 20.427 ± 2.895 and that in the control group was 20.153 ± 3.128. The mean age of patients with osteosarcoma was 15.234 ± 9.051 years, and the male-to-female ratio was 1.6:1. The mean age of patients with chondrosarcoma and Ewing sarcoma was 52.731 ± 11.619 and 15.619 ± 11.910 years, and the male-to-female ratio was approximately 2:1 and 1:1, respectively. In addition, the number of patients with UPS was 65.800 ± 4.550. After a follow-up of 16.457 ± 5.014 months, the status of all patients was compiled statistically.

**Table 2:** The demographic characteristics of experimental group and control group (n = 116).

Characteristics				Experimental group (n = 59)	Control group (n = 57)	*t*/χ^2^	*P*
Age				25.474 ± 20.776	25.068 ± 19.560	0.108	.914
Sex	Male			34 (57.627)	35 (61.404)	0.172	.679
	Female			25 (42.373)	22 (38.596)		
Family history	Yes			7 (11.864)	5 (8.772)	0.299	.585
	No			52 (88.136)	52 (91.228)		
BMI				20.427 ± 2.895	20.153 ± 3.128	0.607	.545
Smoking	Yes			12 (20.339)	10 (17.544)	0.147	.701
	No			47 (79.661)	47 (82.456)		
Drinking	Yes			14 (23.729)	17 (29.825)	0.550	.458
	No			45 (76.271)	40 (70.175)		
Pathology	Osteosarcoma			30 (50.847)	34 (59.649)	2.218	.528
	Chondrosarcoma			14 (23.729)	12 (21.053)		
	Ewing sarcoma			11 (18.644)	10 (17.544)		
	Undifferentiated pleomorphic sarcoma (UPS)			4 (6.780)	1 (1.754)		
Locations	Right femur			15 (25.424)	14 (24.561)	3.464	.326
	Left femur			22 (37.288)	27 (47.369)		
	Right tibia			6 (10.169)	8 (14.035)		
	Left tibia			16 (27.119)	8 (14.035)		
Preoperative chemotherapy	Osteosarcoma		Methotrexate + Doxorubicin + Cisplatin/ + Ifosfamide	30 (50.847)	34 (59.649)	2.098	.350
	Ewing sarcoma		Vincristine + Doxorubicin + Cyclophosphamide/ Ifosfamide + Etoposide	11 (18.644)	10 (17.544)		
	UPS		Doxorubicin + Ifosfamide	4 (6.780)	1 (1.754)		
Postoperative adverse reactions		Yes		8 (13.559)	9 (15.789)	0.115	.734
		No		51 (86.441)	48 (84.211)		
Following up				16.647 ± 5.546	16.441 ± 4.489	0.035	.972
Status	Dead			6 (10.169)	12 (21.053)	2.619	.106
	Living			53 (89.831)	45 (78.947)		

BMI = body mass index, UPS = undifferentiated pleomorphic sarcoma.

The results showed no significant associations between age (*P* = .914), sex (*P* = .679), family history (*P* = .585), and BMI (*P* = .545) or between the experimental and control groups. Also, there were no differences between groups at smoking history (*P* = .701), drinking history (*P* = .458), pathology (*P* = .528), and locations (*P* = .326), as well as no differences were observed between pre-operation chemotherapy (*P* = .350), adverse reactions in post-operation (*P* = .734), following-up (*P* = .972) and status (*P* = .106) and experimental group and control group. The results indicated that the data in the experimental and control groups had a strong comparability.

### 3.2. Effects of whole-course nursing

**Figure 2.**
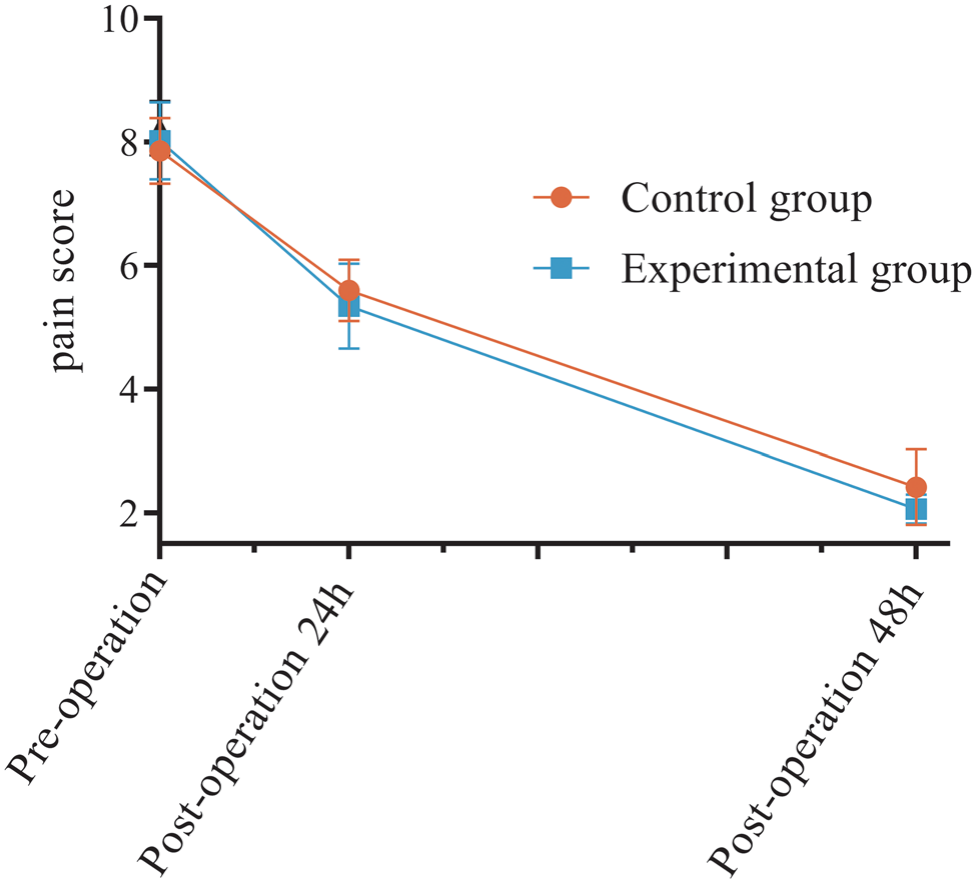
Line chart illustrating changed in pain scores in the control group (n = 59) and experimental group (n = 57) at pre-operation and post-operation 24 hours and 48 hours postoperatively. Data were expressed as mean ± standard deviation (SD). Error bars represented SD. SD = standard deviation.

As shown in Table 3, the difference between the pain indices of the 2 groups was not statistically significant (*P* = .089), but 24 hours after surgery, the pain scores of the 2 groups were significantly reduced (Fig. 2), and the pain indices of patients in the whole-course nursing group were lower than those in the normal nursing group (*P* = .046). Furthermore, 48 hours after surgery, the pain indexes in the whole course nursing group were significantly reduced (Fig. 2) and much lower than those in the control group (*P* < .001).

**Table 3:** The comparison of pain score, functional scale, satisfaction with pain relief and nursing, adverse emotion assessment and life quality score in experimental group and control group (n = 116).

Characteristics			Experimental group (n = 59)	Control group (n = 57)	*t*	*P*
Pain score		Pre-operation	8.051 ± 0.600	7.874 ± 0.502	1.717	.089
		Post-operation 24h	5.271 ± 0.448	5.456 ± 0.537	−2.017	**.046**
		Post-operation 48h	2.085 ± 0.281	2.421 ± 0.596	−3.908	**<.001**
Lower extremity functional scale	Functional activities	Pre-nursing	15.780 ± 3.327	14.965 ± 4.132	1.172	.244
		Post-nursing	22.186 ± 3.082	20.175 ± 4.322	**2.893**	**.006**
	Stepping ability	Pre-nursing	15.238 ± 4.283	15.813 ± 3.560	−0.610	.543
		Post-nursing	23.661 ± 3.335	20.368 ± 4.337	**4.593**	**<.001**
	Limb pain	Pre-nursing	23.754 ± 1.199	24.034 ± 1.017	−1.356	.178
		Post-nursing	12.288 ± 2.060	13.491 ± 2.543	−**2.804**	**.006**
	Gait changing	Pre-nursing	17.632 ± 3.778	18.339 ± 3.813	−1.003	.318
		Post-nursing	21.509 ± 3.115	19.544 ± 2.666	**3.644**	**<.001**
Satisfaction with pain relief			8.085 ± 0.651	7.123 ± 0.758	**7.343**	**<.001**
Satisfaction with nursing			95.186 ± 2.576	93.158 ± 3.945	**3.290**	**.001**
Adverse emotion assessment			14.160 ± 1.752	15.772 ± 2.196	−**3.155**	**.002**
Quality of life score		Pre-nursing	43.915 ± 5.873	43.579 ± 5.946	0.306	.760
		Post-nursing	87.009 ± 8.766	80.570 ± 13.462	**3.063**	**.003**

Bold values represent the *t*-values for comparisons between different groups.

**Figure 3.**
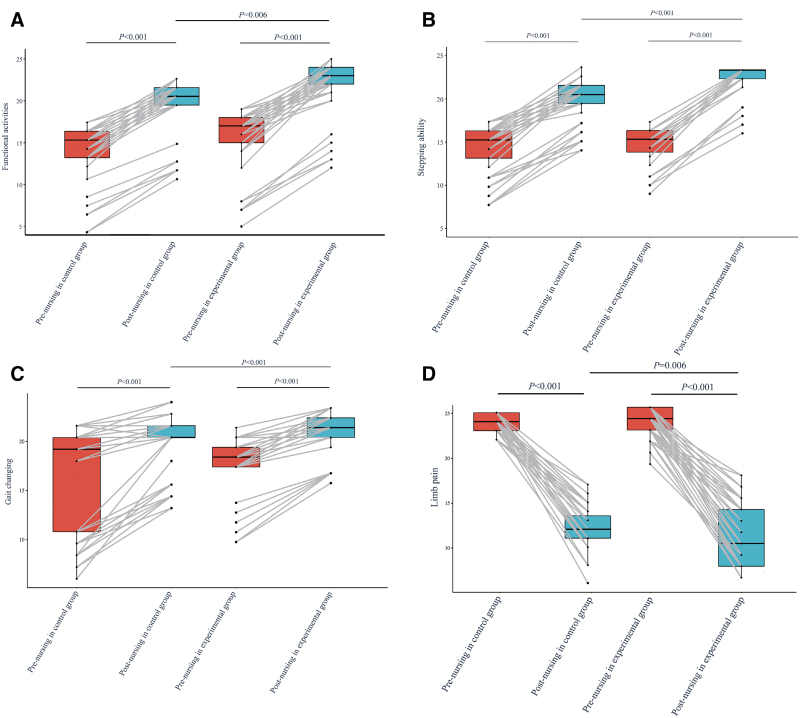
Histogram of patients’ functional activities, stepping ability, limb pain and gait changing in pre-operation and post-operation in experimental group (n = 57) and control group (n = 59), assessed by the Lower Extremity Functional Scale (LEFS). Data were presented as mean ± SD; error bars represented SD. (A) The functional activities. (B) The stepping ability. (C) The limb pain. (D) The gait changing. LEFS = Lower Extremity Functional Scale, SD = standard deviation.

For the lower extremity functional scale, the differences between functional activities (*P* = .244), stepping ability (*P* = .543), limb pain (*P* = .178), and gait change (*P* = .318) between the 2 groups were not statistically significant. After the whole-course nursing, functional activities (Fig. 3A: *P* = .006), stepping ability (Fig. 3B: *P* < .001), and gait change (Fig. 3C: *P* < .001) were significantly improved compared to those in normal rehabilitation nursing. In addition, limb pain scores in the experimental group were significantly lower than those in the control group (Fig. 3D, *P* = .006).

**Figure 4.**
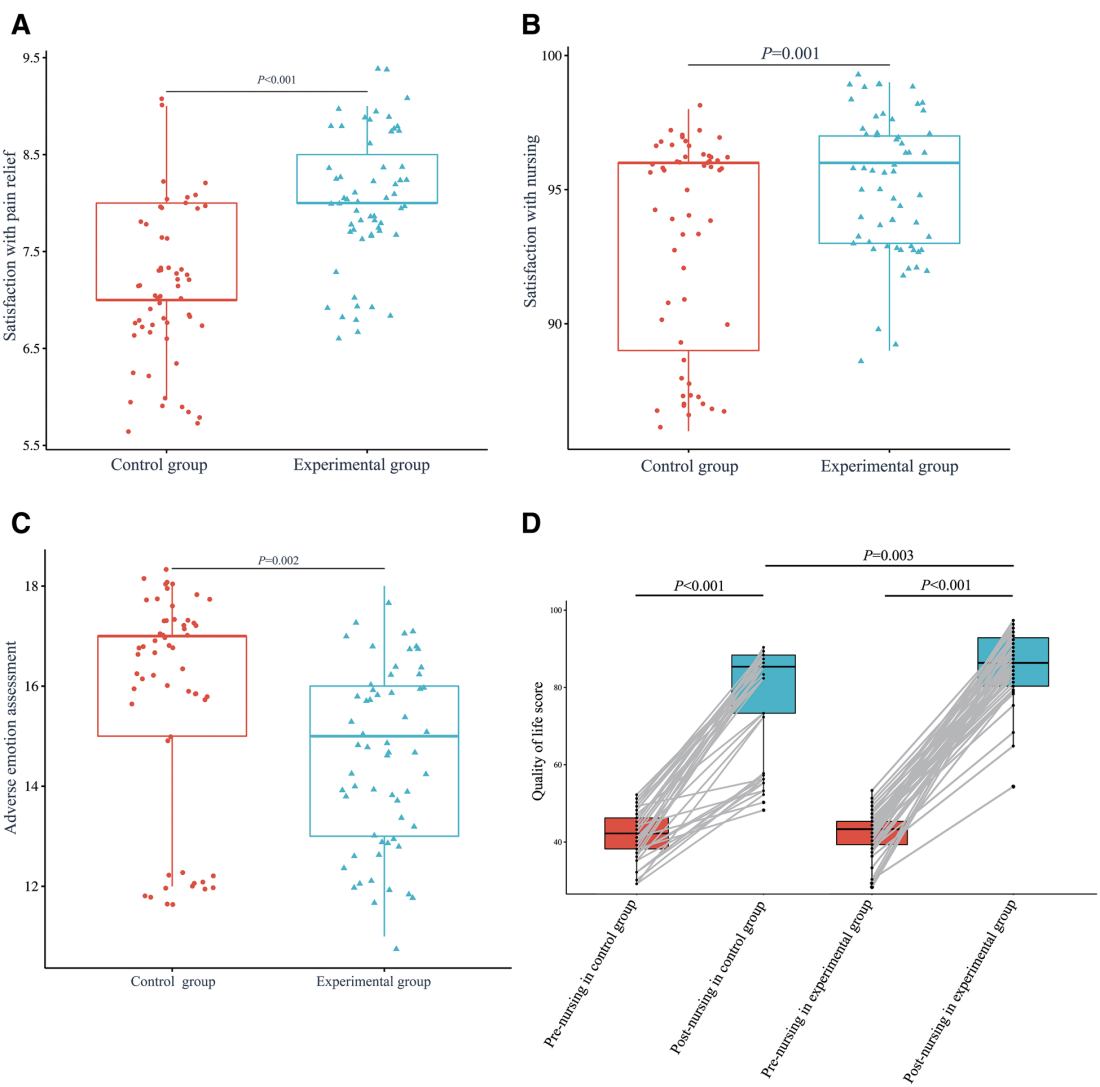
Histogram of comparing subjective outcomes between groups (control: n = 59; experimental: n = 57). Data were expressed as mean ± SD; error bars represented SD. (A) Satisfaction with pain relief assessed by HPOI. (B) Satisfaction with nursing care assessed by hospital-designed questionnaire at discharge. (C) Adverse emotion assessment assessed by HAMA scale. (D) Quality of life assessed by Karnofsky index. HAMA = Hamilton Anxiety Scale, HPOI = Houston Pain Outcome Instrument, Karnofsky index = Karnofsky Performance Status Index, SD = standard deviation.

The HOPI questionnaire results revealed that all patients were satisfied with pain relief, but the satisfaction index of the experimental group was significantly higher than that of the control group (Fig. 4A, *P* < .001). Similarly, both of 2 groups with nursing satisfaction scores reached “very satisfied,” but the satisfaction scores of the experimental group was still significantly higher than that of the control group (Fig. 4B: *P* = .001).

After rehabilitation nursing, the adverse emotion scores of patients were more relieved, but compared with the control group, the whole-process nursing group patients scored lower on adverse emotions (Fig. 4C: *P* = .002) and higher quality of life (Fig. 4D: *P* = .003).

### 3.3. Intervention outcomes

In the experimental group, a total of 8 patients had postoperative adverse complications, accounting for 13.559%, among which 2 patients had delayed wound healing, 2 patients had infection, and 4 patients had local bone absorption on the prosthesis contact surface. In the control group, 9 patients had postoperative complications, accounting for 15.789%, of which 2 patients had pulmonary metastasis, 3 patients had superficial incision infection, and 4 patients had local recurrence.

**Figure 5.**
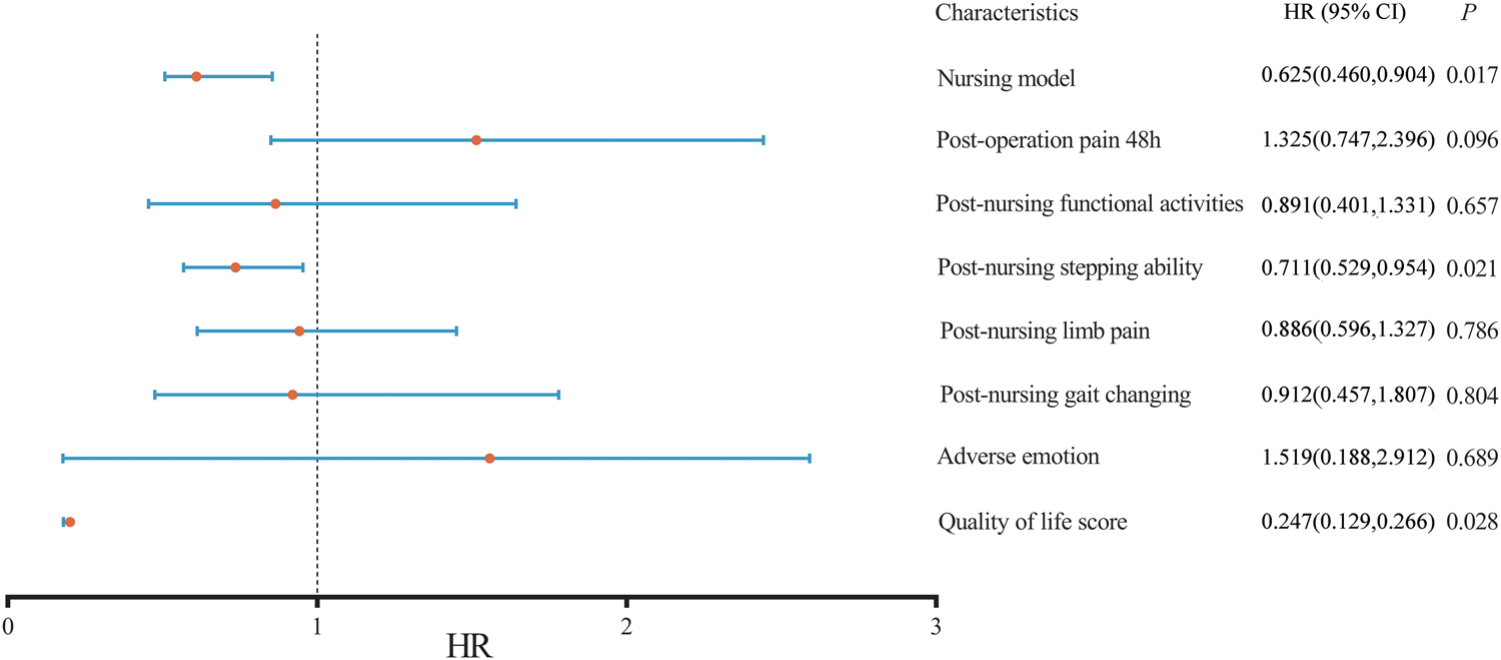
Forest plot of Cox proportional hazards regression analysis identifying independent prognostic factors for postoperative outcomes. Hazard ratios (HR) were presented with corresponding 95% confidence intervals (CI) and *P*-values. CI = confidence interval, HR = hazard ratio.

As shown in Figure 5 and Table 4, different nursing models (*P* = .017), post-operation (*P* = .021), and quality of life scores (*P* = .028) were independent factors in the prognosis of malignant bone and soft tumors of the lower limbs.

**Table 4:** Cox regression analyses of nursing model, post-operation pain, post-operation functional activities, stepping ability, limb pain, gain changing, adverse emotion, and quality of life.

Characteristics	β	SE	Wald	Exp β (95% CI)	*P*
Nursing model	0.159	0.158	1.886	0.609 (0.507, 0.854)	**.017**
Post-operation pain 48h	0.922	0.554	2.774	1.514 (0.850, 2.442)	.096
Post-nursing functional activities	−0.145	0.327	0.197	0.865 (0.455, 1.642)	.657
Post-nursing stepping ability	−0.306	0.132	5.358	0.736 (0.568, 0.954)	**.021**
Post-nursing limb pain	−0.060	0.220	0.074	0.942 (0.612, 1.450)	.786
Post-nursing gait changing	0.084	0.337	0.061	0.920 (0.475, 1.780)	.804
Adverse emotion	0.443	1.105	0.161	1.557 (0.178, 2.591)	.689
Quality of life score	−0.183	0.290	0.398	0.201 (0.180, 0.210)	**.028**

CI = confidence interval, SE = standard error.

Bold values represent the *t*-values for comparisons between different groups.

## 4. Discussion and conclusions

The typical symptom of patients with malignant bone tumors is pain, which can cause anxiety, depression, or other negative emotions, and these negative emotions would aggravate the deterioration of the tumors.^[9]^ The diagnosis of osteosarcoma has a significant psychological impact on patients. Negative emotions are exacerbated by the toxic side effects of preoperative neoadjuvant chemotherapy, such as nausea, vomiting, hair loss, and the high cost of treating malignant bone tumors. Postoperative complications are complicated, and functional recovery and chemotherapy times are relatively long, which can cause patients to lose confidence in treatment, further aggravate anxiety, and reduce compliance, all of which are detrimental to recovery.^[10]^ Whole-course nursing provides comprehensive nursing intervention in the treatment process of patients from multiple aspects, evaluates emotional problems, reduces negative emotions, mobilizes initiative, improves initiative and enthusiasm, and promotes the rehabilitation of patients. We conducted a rigorous randomized controlled study on normal nursing and whole-course nursing interventions for different patients and found that whole-course nursing intervention had a significant positive effect on relieving pain, promoting postoperative functional recovery, and improving the quality of life and prognosis of patients.

In this study, the pain indices of patients were all high before surgery, and then decreased 24 hours and 48 hours after surgery, regardless of routine nursing or whole-course nursing. However, the patients’ pain indices receiving whole-course nursing were significantly lower than those of patients receiving normal nursing, and patient satisfaction in the whole-course nursing group was significantly higher than that of the normal nursing group. Our findings aligned with those of previous studies that have demonstrated the positive impact of comprehensive nursing interventions on postoperative recovery and emotional well-being. For example, a study conducted by Xiao-Li et al (2021) revealed that targeted pain management nursing significantly reduced Visual Analog Scale (VAS) pain scores after orthopedic surgery, thereby supporting our observation that continuous, holistic nursing care contributes to reduced postoperative pain and enhanced patient satisfaction.^[11]^ Furthermore, our study found that whole-course nursing intervention had significant advantages in relieving postoperative pain and improving patient satisfaction and nursing quality, which has strong clinical application value. Given the aforementioned benefits of whole-course nursing, the results suggest that for lower limb patients with malignant bone tumors, targeted whole-course nursing psychological intervention could effectively reduce patients’ pain, improve pain relief, and have a positive effect on patient prognosis.

Pain caused by the tumor, anxiety regarding treatment, and poor prognosis are the major causes of adverse emotions in patients with malignant bone tumors. In whole-course nursing, a targeted nursing plan formulated according to the patients’ actual situation can ensure the quality of daily care. Appropriate conversation and encouragement can help patients maintain positive attitudes during hospitalization. Timely exercise and encouragement based on the patient’s actual situation after surgery can reduce the incidence of postoperative tissue adhesion complications. Similarly, Ping et al demonstrated that a structured, process-oriented rehabilitation model can significantly enhance lower limb function and improve emotional well-being in patients undergoing limb salvage surgery for bone tumors.^[12]^ These findings further support our conclusion that continuous and individualized nursing interventions are essential for optimizing both physical and psychological recovery outcomes in this patient population. Moreover, the quality of life score analysis revealed that the patients’ quality of life was significantly improved with the assistance of whole-course nursing, suggesting that whole-course nursing can effectively alleviate adverse emotions, accelerate compliance, maximize the therapeutic effect, greatly improve the quality of life, and have a significant positive effect on patient prognosis. Engagement in the whole-course care program may have contributed to better limb function activities.^[13]^ Future studies should demonstrate the clinical application value of whole-course nursing in relaxing adverse emotions, increasing patient compliance, promoting functional recovery of the affected limbs, and improving quality of life.

Whole-course rehabilitation nursing after discharge provides continuous nursing services for patients, effectively compensates for the lack of guidance and supervision of patients in normal nursing, prevents various complications, and significantly improves patient prognosis. In our study, patients in the experimental group received extended nursing after discharge and were supervised and urged to conduct rehabilitation training of affected limb function under the intervention of the whole-course nursing mode. The results demonstrated that the complication rates of the patients were low, demonstrating the effectiveness of extended nursing after discharge to ensure rehabilitation training for patients. Meanwhile, another study revealed that after discharge, a continuous rehabilitation team composed of third-level physicians and responsible nurses was set up to follow up limb function recovery through outpatient visits and telephone calls and further guide rehabilitation exercise.^[13]^

This study has several limitations. First, the possibility of selection bias cannot be ruled out, as all participants were recruited from a high-level cancer center. This may limit the generalizability of the findings to other healthcare settings with differing resources or patient demographics. Second, the follow-up time of this study was short, and the impact of intervention measures on prognosis was analyzed only in the early and middle stages. Continuous follow-up should be carried out to further analyze the impact of 5-year survival and 10-year survival of patients. Third, data collection during the follow-up period was conducted via telephone or online platforms rather than through face-to-face interactions. This approach may compromise the accuracy of patient-reported outcomes, such as satisfaction, emotional status, and functional recovery, potentially introducing response bias.

This randomized controlled study demonstrated that whole-course nursing for malignant bone and soft tumors of the lower limbs can effectively relieve pain in the affected limbs and have a significant positive effect on weakening adverse emotions, such as anxiety, irritability, and fear. In addition, the whole-course intervention is prone to accelerate the recovery of the lower extremities and decrease complications. Moreover, the overall survivals of patients were positively associated with whole-course nursing, with high satisfaction. It is believed that existing problems are continuously found and solved through the efforts of medical nursing personnel. The full whole-course nursing management model will better serve clinical medical nursing and patients with malignant bones and soft tumors of the lower limbs.

## Author contributions

**Conceptualization:** Linlin Wang, Wen Tian, Jing Jing, Ming Yin.

**Data curation:** Linlin Wang, Wen Tian.

**Formal analysis:** Yanna Sun.

**Investigation:** Yanna Sun.

**Validation:** Wen Tian.

**Writing – original draft:** Linlin Wang.

**Writing – review & editing:** Wen Tian, Jing Jing, Ming Yin, Yanna Sun.




