# Supplementary material for: The efficacy of whole-course nursing on malignant bone and soft tumors of lower limbs: A randomized study
Source: Medicine (Baltimore). 2026 Aug 7;105(32):e49172. doi: 10.1097/MD.0000000000049172 (PMC13456889; doi:10.1097/MD.0000000000049172)
Supplement: Supplementary file 1 [file medi-105-e49172-s001.docx]

| Supplementary Table S1 Outcome assessment timeline | | | | | |
| --- | --- | --- | --- | --- | --- |
| Assessment Tool | Preoperative | 24h Post-op | 48h Post-op | Discharge | 6-Month Follow-up |
| VAS | √ | √ | √ | × | × |
| LEFS | √ | × | × | √ | × |
| HAMA | × | × | × | √ | × |
| HOS | × | × | × | √ | × |
| Patient Satisfaction | × | × | × | √ | × |
| Adverse Events | × | × | × | × | √ |
| Quality of Life | × | × | × | × | √ |

√ Assessment performed at this time point

× Not assessed at this time point
