# Supplementary material for: The efficacy of whole-course nursing on malignant bone and soft tumors of lower limbs: A randomized study
Source: Medicine (Baltimore). 2026 Aug 7;105(32):e49172. doi: 10.1097/MD.0000000000049172 (PMC13456889; doi:10.1097/MD.0000000000049172)
Supplement: Supplementary file 2 [file medi-105-e49172-s002.docx]

Dear Editor,

I'm delighted to hear from you.

Apologies for our oversight in not uploading the questionnaire related to this study, specifically the professionally designed questionnaire on patient satisfaction with the whole-curse nursing process at our hospital. We have now attached the questionnaire as supplementary material. The first part includes the Chinese version of the questionnaire, followed by its English translation.

Thank you for reminding us and we wish you a happy life once again.

Sincerely,

Linlin Wang

病人对护士工作满意度调查问卷（中文）

尊敬的先生/女士：

您好，为进一步深化本医院“以病人为中心”的护理理念，改革护理人员的工作模式，实施责任制度整体护理，为病人提供全面全程优质护理服务，进一步提高在优质护理活力中护士的护理质量，发现在临床工作中存在的问题，我们特意进行此次调查研究，本研调查研究结果用于指导临床护理工作，属不记名调查，希望能得到您的配合，非常感谢！

（一）一般资料

性别	年龄	职业	居住地区：城市/农村

本次住院天数	住院次数

文化程度：小学□ 初中□ 高中□ 本科□ 研究生□

婚姻状况：未婚□ 已婚□ 丧偶□

（二）填写要求

10分=非常满意；8分=比较满意；6分=满意度一般；4分=比较不满意；2分=不满意；0分=非常不满意。

请在下列各项中符合您的情况的选项打√

1. 您对病区环境满意吗？

A. 10分；B. 8分; C. 6分; D. 4分; E. 2分; F. 0分。

2. 您对责任护士对您的健康教育满意吗？

A. 10分；B. 8分; C. 6分; D. 4分; E. 2分; F. 0分。

3. 您对责任护士的专业技能满意吗？

A. 10分；B. 8分; C. 6分; D. 4分; E. 2分; F. 0分。

4. 您对责任护士的服务态度满意吗？

A. 10分；B. 8分; C. 6分; D. 4分; E. 2分; F. 0分。

5. 您对责任护士的责任态度满意吗？

A. 10分；B. 8分; C. 6分; D. 4分; E. 2分; F. 0分。

6. 您对责任护士的护理及时性满意吗？

A. 10分；B. 8分; C. 6分; D. 4分; E. 2分; F. 0分。

7. 您对责任护士的护理主动性满意吗？

A. 10分；B. 8分; C. 6分; D. 4分; E. 2分; F. 0分。

8. 您对责任护士的护理人性化满意吗？

A. 10分；B. 8分; C. 6分; D. 4分; E. 2分; F. 0分。

9. 您对责任护士与您的沟通情况满意吗？

A. 10分；B. 8分; C. 6分; D. 4分; E. 2分; F. 0分。

10. 您对责任护士的着装仪表情况满意吗？

A. 10分；B. 8分; C. 6分; D. 4分; E. 2分; F. 0分。

再次感谢您对此次调查问卷的支持！

Patient Satisfaction Questionnaire for Nurses (English)

Dear Sir/Madam,

Hello, in order to further enhance the hospital's "patient-centered" nursing concept, reform the working mode of nursing staff, implement the overall nursing responsibility system, provide comprehensive and high-quality nursing services for patients, and further improve the nursing quality of nurses in delivering exceptional care. To identify any existing issues in clinical work, we have conducted this investigation and research. The results of this study will be used to guide clinical nursing practices anonymously.

We kindly request your cooperation and thank you very much!

(1) General information

Gender	Age	Occupation	Area of Residence: Urban/rural

Length of stay	Hospitalizations

Educational level: Primary school □ Middle school □ High school □ Undergraduate □ Postgraduate □

Marital status: Unmarried □ Married □ Widowed □

(2) Filling requirements

10 points = very satisfied; 8 points = quite satisfied; 6 points = average satisfaction; 4 points = less satisfied; 2 points = dissatisfied; 0 points = very dissatisfied.

Please tick the options below that match your situation

1. Are you satisfied with the environment of the ward?

A. 10 points; B. 8 points; C. 6 points; D. 4 points; E. 2 points; F. 0 points.

2. Are you satisfied with your health education from the responsible nurse?

A. 10 points; B. 8 points; C. 6 points; D. 4 points; E. 2 points; F. 0 points.

3. Are you satisfied with the professional skills of the responsible nurse?

A. 10 points; B. 8 points; C. 6 points; D. 4 points; E. 2 points; F. 0 points.

4. Are you satisfied with the service attitude of the responsible nurse?

A. 10 points; B. 8 points; C. 6 points; D. 4 points; E. 2 points; F. 0 points.

5. Are you satisfied with the responsible attitude of the responsible nurse?

A. 10 points; B. 8 points; C. 6 points; D. 4 points; E. 2 points; F. 0 points.

6. Are you satisfied with the timeliness of the nurse's care?

A. 10 points; B. 8 points; C. 6 points; D. 4 points; E. 2 points; F. 0 points.

7. Are you satisfied with the initiative of the responsible nurse?

A. 10 points; B. 8 points; C. 6 points; D. 4 points; E. 2 points; F. 0 points.

8. Are you satisfied with the humanization of the nurse's care?

A. 10 points; B. 8 points; C. 6 points; D. 4 points; E. 2 points; F. 0 points.

9. Are you satisfied with the communication between the responsible nurse and you?

A. 10 points; B. 8 points; C. 6 points; D. 4 points; E. 2 points; F. 0 points.

10. Are you satisfied with the dress and appearance of the responsible nurse?

A. 10 points; B. 8 points; C. 6 points; D. 4 points; E. 2 points; F. 0 points.

Thank you again for your support of this survey!
